# The Band for Preserving the Teeth

**Published:** 1904-06

**Authors:** W. Farley

**Affiliations:** Paris, France.


### THE BAND FOR PRESERVING THE TEETH.

By Dr. W. Farley, Paris, France.

Dentistry, insofar as concerns the preservation of teeth by means of "filling" is a failure in the field of the back teeth when the latter are attacked on a line with or below the gum. It is estimated that the life of an amalgam filling will average four years. Also that four-fifths of all fillings are amalgam. Amalgam, then, can not be called a permanent filling. It is not serious enough for the pretenses of a profession. It is more a barber's material when employed in such cases. This will not do. Let us spend less time in discussing ethics and more time in discussing the preservation of ordinary teeth, which, heretofore, have evaded our boasted skill. If I were addressing a room full of students, I would tell them that a tooth which is decayed below the gum margin cannot be preserved with a plastic filling, seldom with gold, *but,* as a rule, always with a band. Any careful dentist can crown such teeth and preserve them, but this may be going too fast on his part; and, besides, is it always necessary to crown such teeth? I emphatically state that it is better to crown them outright than to fill them, but there is, however, a compromise between crowning and filling, and this compromise is effected by means of a band. Although requiring rather mature skill, such as follows from prolonged experience, this band method for preserving the teeth might be sucessfully practiced after the student has undergone a special training in its adjustment. The *modus operandi* is as follows:

Case, an upper first bicuspid. Separate with a diamond disk, and grind around the tooth sparingly. Fit a band the same as for a gold cap. Trim the articulating edges and draw them in flush to conform to the tooth. Contour the part which is to restore the decayed portion by soldering either a piece of pure gold or platinum of a sufficient thickness to allow the band when in place to *knuckle* against the neighboring tooth. To prevent moving, when permanently fixed, solder a platinum pin, taken from a broken porcelain tooth, inside of the band so that is will project into the cavity. Now cut the buccal part of the band away so as to leave a narrow strip of gold at the gum margin. This exposes most of the remaining portion of the natural crown. The palatal side should be left as high as possible. The band is thus ready to be cemented in place (preliminary treatment anticipated). Wash the tooth with alcohol, dry, mix cement, fill in the cavity, also smear it around the tooth, push the band in position, using a foot-plugger and hand mallet to drive it gently home. Particular care must be exercised in forcing the cement all over. A sure way is to push it up with a pellet of cotton.

In some cases a pivot can be fitted in the root. When this may be found useful, the band can be cut away excepting where it restores the contour broken by decay. Instead of finishing with a cement, amalgam, or gold foil surface, the grinding part may restored with a block of porcelain or pure gold. The best way is to finish with gold foil or amalgam. For operators who have full confidence in their ability, such a band as prescribed can be employed as a matrix in building up the vacuum with gold foil. I prefer, when using the band as a matrix only, to begin with tin cylinders introduced on the wedge system of Dr. Schuyler, of Rochester; using three and continuing with gold cylinders and finishing with cohesive foil.—*Items of Interest.*

